# The CXXC Motifs Are Essential for the Function of BosR in *Borrelia burgdorferi*

**DOI:** 10.3389/fcimb.2019.00109

**Published:** 2019-04-16

**Authors:** Charlotte Mason, Xiaoyan Liu, Spoorthy Prabhudeva, Zhiming Ouyang

**Affiliations:** ^1^Department of Molecular Medicine, University of South Florida, Tampa, FL, United States; ^2^Department of Microbiology, University of Texas Southwestern Medical Center, Dallas, TX, United States

**Keywords:** Lyme disease, spirochetes, *Borrelia burgdorferi*, gene expression, gene regulation, pathogenesis

## Abstract

BosR, a Fur family member, is essential for the pathogenesis of the Lyme disease pathogen, *Borrelia burgdorferi*. Unlike typical Fur proteins in which DNA binding represses gene expression, binding of BosR to the *rpoS* promoter directly activates *rpoS* transcription in *B. burgdorferi*. However, virtually nothing is known concerning potential structural features and amino acid residues of BosR that are important for protein function and virulence regulation in *B. burgdorferi*. Particularly, it remains unknown what structural motifs or residues of BosR coordinate Zn, although previous analyses have indicated that the function of BosR may depend on Zn. To address these information gaps, we herein introduced mutations into four conserved cysteine residues in two putative CXXC motifs of BosR. Our data showed that the ability of BosR to bind Zn was dramatically reduced when the CXXC motifs were mutated. Moreover, we found that the two CXXC motifs contributed to the ability of BosR to form dimers. By using a *trans*-complementation genetic approach, we additionally demonstrated that both CXXC motifs of BosR were essential for *in vivo* gene expression regulation. Mutation of any of the four cysteines abolished the transcriptional activation of *rpoS*. In contrast to wild type BosR, each mutant protein was incapable of binding the *rpoS* promoter in electrophoretic mobility shift assays. The combined data strongly support that the two CXXC motifs and four cysteines constitute the structural site essential for Zn-coordination, protein dimerization, and the unique regulatory activity of BosR.

## Introduction

Lyme disease, caused by the spirochete *Borrelia burgdorferi*, is the most prevalent vector-borne disease in the United States (Mead, 2015; Rosenberg et al., 2018). *B. burgdorferi* survives in nature through a complex enzootic life cycle involving an arthropod tick vector and mammalian hosts like small rodents (Burgdorfer et al., 1982; Steere, 1993; De Silva and Fikrig, 1995; Schwan, 1996; Steere et al., 2004, 2016). During its transit between tick and animals, *B. burgdorferi* undergoes dramatic adaptive changes in order to survive in the continuously changing environments (Schwan et al., 1995; Liang et al., 2002, 2004; Pal and Fikrig, 2003; Samuels, 2011; Radolf et al., 2012). Host adaptation of *B. burgdorferi* is usually achieved through differential gene expression, which is largely controlled by transcriptional regulatory factors such as the alternative sigma factor RpoS (also called σ^S^). In addition to directly activating the expression of lipoproteins required for mammalian infection such as OspC and DbpA, RpoS also indirectly represses expression of the tick colonization factor OspA (Pal et al., 2000, 2004, 2008; Hübner et al., 2001; Alverson et al., 2003; Caimano et al., 2004, 2005, 2007; Eggers et al., 2004; Yang et al., 2004, 2005; Gilbert et al., 2007; Ouyang et al., 2008, 2010; Drecktrah et al., 2013). In *B. burgdorferi*, transcription of *rpoS* is promoted by another alternative sigma factor σ^54^ (i.e., RpoN) via a classical −24/−12 promoter (Fisher et al., 2005; Burtnick et al., 2007; Lybecker and Samuels, 2007; Smith et al., 2007). Like all other bacterial σ^54^ systems, activation of σ^54^-dependent *rpoS* transcription in *B. burgdorferi* requires an AAA+ activator ATPase called Rrp2 (Yang et al., 2003; Burtnick et al., 2007; Boardman et al., 2008; Ouyang et al., 2008, 2014b; Blevins et al., 2009; Groshong et al., 2012; Yin et al., 2016; Ouyang and Zhou, 2017). Although the mechanism remains obscure, it is believed that the ATPase activity of Rrp2 triggers the conversion of an inactive closed complex (formed by σ^54^ and the *rpoS* promoter) to an active open complex. Recent studies showed that, however, different from other σ^54^ systems, Rrp2 alone is not enough for activating *rpoS* transcription in *B. burgdorferi*. Rather, another activator called BosR is crucially required for σ^54^-dependent *rpoS* transcription (Hyde et al., 2009, 2010; Ouyang et al., 2009b, 2011; Katona, 2015). *rpoS* transcription was abolished when *bosR* was deleted from *B. burgdorferi* but restored when *bosR* mutation was complemented. Furthermore, BosR strongly binds to BS1 and BS2 sites in the *rpoS* promoter region via a novel DNA element termed BosR box (Ouyang et al., 2011, 2014a), supporting that BosR directly binds to the *rpoS* promoter and activates gene expression.

Sequence homology suggests that BosR belongs to the Fur protein family (Fraser et al., 1997; Boylan et al., 2003; Katona et al., 2004). In a wide variety of microorganisms, Fur family members are known principally as metal-responsive DNA-binding transcriptional regulators (Hantke, 1981, 2001; Carpenter et al., 2009; Jacquamet et al., 2009; Troxell and Hassan, 2013; Fillat, 2014). Fur proteins consist of two structural domains including an N-terminal winged helix-turn-helix DNA-binding domain and a C-terminal dimerization domain, connected by a flexible hinge region. Although Fur also influences the expression of genes unrelated to metal transport, the Fur family is noted for regulating genes involved in the homoeostasis of transition metal ions such as Fe, Zn, Mn, and Ni (Hantke, 1981, 2001; Carpenter et al., 2009; Jacquamet et al., 2009; Troxell and Hassan, 2013; Fillat, 2014). Structural analyses of numerous Fur proteins have revealed two sites involved in metal binding (Pohl et al., 2003; Lee and Helmann, 2006; Pecqueur et al., 2006; Traoré et al., 2006, 2009; Lucarelli et al., 2007; Ahmad et al., 2009; An et al., 2009; Carpenter et al., 2009; Jacquamet et al., 2009; Sheikh and Taylor, 2009; Davies et al., 2011; Dian et al., 2011; Shin et al., 2011; Butcher et al., 2012; Makthal et al., 2013; Troxell and Hassan, 2013; Fillat, 2014; Gilston et al., 2014; Lin et al., 2014; Deng et al., 2015). The first site (S1) is located in the dimerization domain. Usually occupied by a Zn^2+^, the S1 site is critical for protein dimerization and thus called the structural site. The second metal binding site (S2) is located in the hinge region between the DNA-binding domain and the dimerization domain. It has been established that the metal ion (e.g., Fe^2+^, Mn^2+^, Zn^2+^, or Ni^2+^) occupying the S2 site determines the specific regulatory function of Fur protein. Thus, the S2 site is called the regulatory site. On rare occasions, Fur proteins such as those in *Helicobacter pylori* and *Campylobacter jejuni* contain a third metal binding site (S3) in the dimerization domain, which is also important for the regulatory function of protein (Dian et al., 2011; Butcher et al., 2012). In general, in absence of metal cofactors, the DNA-binding domain of Fur employs an “open” conformation that is incapable of DNA binding. Occupancy of S1 with a Zn^2+^ induces Fur dimerization, which brings the two DNA-binding domains (of two monomers) into closer proximity. Binding of metal ions to S2 further triggers significant conformational changes, which in turn stimulates the formation of a “closed” DNA-binding domain. As a result, the “closed” Fur binds to promoter DNA with high affinity and represses gene expression.

Like other Fur proteins, BosR was predicted to consist of an N-terminal DNA-binding domain and a C-terminal dimerization domain (Fraser et al., 1997; Boylan et al., 2003; Katona et al., 2004; Ouyang et al., 2011). Different from the classical paradigm that DNA-binding of Fur results in gene repression, BosR directly binds to the *rpoS* promoter and activates σ^54^-dependent *rpoS* transcription in *B. burgdorferi* (Ouyang et al., 2011, 2014a, 2016). BosR also functions as an auto-activator in *B. burgdorferi* (Ouyang et al., 2016). Moreover, BosR avidly binds Zn, suggesting that the function of BosR is dependent on Zn (Boylan et al., 2003; Katona et al., 2004; Ouyang et al., 2011; Wang et al., 2013). However, the potential structure-function relationship of BosR heretofore has not been characterized, and the structural bases coordinating Zn remain unknown. This represents a critical information gap given the fact that BosR functions as a unique transcriptional activator in *B. burgdorferi*. Herein, we analyzed the contributions of two CXXC structural motifs of BosR to Zn binding and protein dimerization through site-directed mutagenesis. We also examined the roles of these motifs and amino acid residues in the overall function of BosR, including binding to the *rpoS* promoter and activation of *rpoS* expression.

## Materials and Methods

### Bacterial Strains and Culture Conditions

All strains and plasmids used in this study are described in Table 1. Infectious *B. burgdorferi* strain 297 (Hughes et al., 1992) was used as wild type (WT) strain throughout this study. The isogenic *bosR* mutant OY08 was constructed in our previous study (Ouyang et al., 2011). *B. burgdorferi* was routinely cultured at 37°C and 5% CO_2_ in BSK-II medium (Pollack et al., 1993) supplemented with 6% rabbit serum (Pel-Freeze, Rogers, AR). When appropriate, supplements were added to media at following concentrations: kanamycin, 160 μg/ml; streptomycin, 100 μg/ml. Growth of *B. burgdorferi* was monitored by dark-field microscopy. *Escherichia coli* strains were cultured in lysogeny broth (LB) medium supplemented with appropriate antibiotics at the following concentrations: ampicillin, 100 μg/ml; kanamycin, 50 μg/ml; or spectinomycin, 100 μg/ml. *E. coli* strain TOP10 (Thermo Fisher Scientific, Grand Island, NY) was used as the cloning host for plasmid construction. All plasmid constructs were confirmed by using PCR amplification, restriction digestion, and sequence analysis.

**Table 1:** Strains and plasmids used in this study.

**Strain or plasmid**	**Description**	**Source**
***B. burgdorferi***		
297	infectious, low-passage *B. burgdorferi*	Hughes et al., 1992
OY08	*B. burgdorferi* 297, Δ*bosR*::Kan	Ouyang et al., 2011
OY110	OY08 transformed with pOY152 (WT BosR)	This study
OY193	OY08 transformed with pOY323 (C114S BosR)	This study
OY194	OY08 transformed with pOY324 (C117S BosR)	This study
OY195	OY08 transformed with pOY325 (C153S BosR)	This study
OY196	OY08 transformed with pOY326 (C156S BosR)	This study
OY197	OY08 transformed with pOY327 (C114S/C153S BosR)	This study
OY198	OY08 transformed with pOY328 (C114S/C156S BosR)	This study
OY357	OY08 transformed with pOY536 (C114D BosR)	This study
OY358	OY08 transformed with pOY537 (C117D BosR)	This study
OY369	OY08 transformed with pOY548 (C153D BosR)	This study
OY370	OY08 transformed with pOY549 (C156D BosR)	This study
***E. coli***		
TOP10	F*^−^ mcrA Δ(mrr-hsdRMS-mcrBC) f80lacZΔM15 ΔlacX74 recA1 araD139 Δ(ara-leu)7697 galU galK rpsL (Str^*R*^) endA1 nupG*	Invitrogen
M15 (pREP4)	F^−^, *Φ80ΔlacM15, thi, lac-, mtl-, recA+, KmR*	Qiagen
**Plasmids**		
pOY99.2	*B. burgdorferi*/*E. coli* shuttle vector with P*flgB-aadA*	Ouyang et al., 2011
pOY152	*bosR* cloned into pOY99.2 at NdeI and BglII sites; plasmid used to complement *bosR* mutation in *trans*.	This study
pOY323	pOY152, C114S BosR	This study
pOY536	pOY152, C114D BosR	This study
pOY324	pOY152, C117S BosR	This study
pOY537	pOY152, C117D BosR	This study
pOY325	pOY152, C153S BosR	This study
pOY548	pOY152, C153D BosR	This study
pOY326	pOY152, C156S BosR	This study
pOY549	pOY152, C156D BosR	This study
pOY327	pOY152, C114S/C153S BosR	This study
pOY328	pOY152, C114S/C156S BosR	This study
pQE30	protein overexpression vector	Qiagen
pOY436	BosR-StrepII cloned into pQE30 at EcoRI and BamHI	This study
pOY496	pOY436, C114S BosR-StrepII	This study
pOY497	pOY436, C117S BosR-StrepII	This study
pOY498	pOY436, C153S BosR-StrepII	This study
pOY499	pOY436, C156S BosR-StrepII	This study
pOY500	pOY436, C114S/C153S BosR-StrepII	This study
pOY501	pOY436, C114S/C156S BosR-StrepII	This study

### Vector Construction for Inducible *bosR* Expression in *B. burgdorferi*

To manipulate *bosR* expression in *B. burgdorferi*, an IPTG-inducible *bosR* expression construct was created by using a *lac*-based inducible expression system (Blevins et al., 2007). First, *bosR* was amplified from strain 297 via PCR using primers ZM113F and ZM195 (Table 2). Purified PCR product was digested with restriction enzymes NdeI and BglII, and then cloned into pOY99.2 (Ouyang et al., 2011) digested with same enzymes. In the resultant shuttle vector pOY152, *bosR* expression was directly controlled by the IPTG-inducible T5 promoter (PpQE30) of the protein overexpression vector pQE30 (Qiagen, Valencia, CA).

**Table 2:** Oligonucleotide primers used in this study.

**Primer**	**Sequence, 5^**′**^-3^**′**^**
**PCR and cloning**	
113F	AATCATATGAACGACAACATAATAGACGTA
ZM195.2	GGCAGATCTTCATAAAGTGATTTCCTTGTTCTCAT
495F	CAGAATTCATTAAAGAGGAGAAATTAACTATGAACGACAACATAATAGACGTA
495.2R	GCGGATCCTCATTTCTCGAACTGCGGGTGGCTCCATAAAGTGATTTCCTTGTTCTCAT
***bosR*** **site-directed mutagenesis**	
C114S forward	TGGCTTCCACAATAGCTCACTTTAAA**A**GCAATAAATGCAATCAAG
C114S reverse	CTTGATTGCATTTATTGC**T**TTTAAAGTGAGCTATTGTGGAAGCCA
C114D forward	TTGGCTTCCACAATAGCTCACTTTAAA**GAT**AATAAATGCAATCAAGTCCACCCTATT
C114D reverse	AATAGGGTGGACTTGATTGCATTTATT**ATC**TTTAAAGTGAGCTATTGTGGAAGCCAA
C117S forward	CCACAATAGCTCACTTTAAATGCAATAAA**A**GCAATCAAGTCCACC
C117S reverse	GGTGGACTTGATTGC**T**TTTATTGCATTTAAAGTGAGCTATTGTGG
C117D forward	TGGCTTCCACAATAGCTCACTTTAAATGCAATAAA**GAT**AATCAAGTCCACCCTATTC
C117D reverse	GAATAGGGTGGACTTGATT**ATC**TTTATTGCATTTAAAGTGAGCTATTGTGGAAGCCA
C153S forward	ACAAAATCTATTGAAATCATTTACTCAGGGCAT**A**GCAATAATTGCTACAAAAA
C153S reverse	TTTTTGTAGCAATTATTGC**T**ATGCCCTGAGTAAATGATTTCAATAGATTTTGT
C153D forward	ACAAAATCTATTGAAATCATTTACTCAGGGCAT**GAT**AATAATTGCTACAAAAA
C153D reverse	TTTTTGTAGCAATTATT**ATC**ATGCCCTGAGTAAATGATTTCAATAGATTTTGT
C156S forward	ATCATTTACTCAGGGCATTGCAATAAT**A**GCTACAAAAAAGATACCC
C156S reverse	GGGTATCTTTTTTGTAGC**T**ATTATTGCAATGCCCTGAGTAAATGAT
C156D forward	ATCATTTACTCAGGGCATTGCAATAAT**GAT**TACAAAAAAGATACCC
C156D reverse	GGGTATCTTTTTTGTA**ATC**ATTATTGCAATGCCCTGAGTAAATGAT

*Restriction enzymes sites are underlined; mutation nucleotides are in bold*.

### Vector Construction for BosR Overexpression in *E. coli*

To obtain recombinant BosR (rBosR), a BosR overexpression construct pOY436 was created, in which *bosR* was fused with a C-terminal Strep-II tag. To this end, *bosR* was amplified from strain 297 by using primers ZM495F and ZM495.2R (Table 2). Specifically, ZM495F contains a 32-bp sequence of pQE30 [including an EcoRI site, the ribosomal binding sequence (RBS), and the ATG start codon] followed by 21-bp of 5′ of *bosR*, whereas ZM495.2R contains a BamHI site and sequences encoding a Strep-II tag (WSHPQFEK). The PCR product was digested with EcoRI and BamHI. Purified DNA was then cloned into pQE30 (Qiagen) digested with same enzymes. In the resultant construct pOY436, *bosR* expression was directly driven by the T5 promoter PpQE30.

### Generation of Site-Directed Mutants of *bosR*

Point mutations of *bosR* were created through site-directed mutagenesis using the QuikChange II XL Site-Directed Mutagenesis Kit (Agilent Technologies, Santa Clara, CA) according to the manufacturer's protocol. The IPTG-inducible *bosR* expression construct pOY152 or the rBosR overexpression vector pOY436 was used as template for the creation of C114S, C114D, C117S, C117D, C153S, C153D, C156S, and C156D mutations. To generate C114S/C153S and C114S/C156S double mutations, pOY323 or pOY496 was used as template for the mutagenesis. The primers used for site-directed mutagenesis are listed in Table 2. The introduced mutations were confirmed by DNA sequencing analysis.

### Induction of BosR by IPTG in *B. burgdorferi*

To complement *bosR* mutation, the shuttle vector pOY152 and its CXXC mutant derivatives were introduced into the *bosR* mutant OY08 through electroporation. *B. burgdorferi* transformation was carried out as described (Samuels, 1995; Samuels et al., 2018). Transformants were selected based on antibiotics resistance. The presence of the shuttle vector in the transformants were verified by using PCR amplification as well as by recovery of the shuttle vector from the complemented strains as we described before (Ouyang et al., 2010).

To induce BosR synthesis, *B. burgdorferi* was inoculated at 10,000 spirochetes per ml into BSK-II medium containing 0-, 50-, 100-, or 200-μM of IPTG. Bacterial growth was monitored by dark-field microscopy. At Day 7 post-inoculation, all cultures reached late-log phase (i.e., ~10^8^ spirochetes per ml). Cells were then harvested via centrifugation for further analyses.

### Overexpression and Purification of Recombinant Proteins

rBosR overexpression construct was transformed into *E. coli* strain M15 (pREP4) (Qiagen). After induction with 1 mM IPTG (Sigma Chemical Co., St. Louis, MO) at 16°C for 16 h, cells were collected through centrifugation and recombinant Strep-II tagged protein was purified using Strep*-*Tactin resins (Qiagen) under native conditions according to the manufacturer's instruction. Eluted protein was concentrated and buffer exchanged with buffer A (20 mM Hepes, 300 mM NaCl, 100 mM L-arginine, 1 mM TCEP [tris(2-carboxyethyl)phosphine], pH 7.0) in an Amicon ultracentrifuge filter device (Millipore, Burlington, MA) with a 10,000 Da cutoff. The concentrated protein was applied to a HiLoad 16/60 Superdex 200 prep grade column and purified on an Äkta fast performance liquid chromatography (FPLC) system (GE Healthcare, Chicago, IL) using buffer A. Subsequent to elution, peak fractions were analyzed by SDS-PAGE and Western Blot. Fractions containing pure rBosR with a homogeneity >95% were pooled and used for further analyses. The molecular weight of rBosR was determined by size exclusion chromatography, using a calibration curve made with gel filtration protein standards including cytochrome c, carbonic anhydrase, bovine serum albumin, alcohol dehydrogenase, and β-amylase (Sigma). Protein concentration was determined by using the BCA protein assay kit (Thermo Fisher Scientific). Mutant versions of rBosR were purified in the same manner as WT rBosR.

### Metal Content Analysis

Metal contents in protein or buffer solutions (as references) were measured by using ICP-AES, at the Research Analytical Laboratory, University of Minnesota. The procedures for protein preparation and metal measurement were essentially as previously described (Ouyang et al., 2009a, 2011).

### SDS-PAGE and Immunoblot Analysis

SDS-PAGE and immunoblot analysis were carried out as previously described (Ouyang et al., 2009b, 2011). In brief, after careful enumeration of spirochetes via dark field microscopy, spirochetes were collected via centrifugation and washed three times with saline water. Pellets were suspended in SDS sample buffer at a concentration of 2 × 10^6^ spirochetes per microliter and boiled for 5 min. A volume of whole cell lysate equivalent to 4 × 10^7^ spirochetes was then loaded per lane on a 12.5% acrylamide gel. Resolved proteins were stained with Coomassie brilliant blue or transferred to nitrocellulose membrane for immunoblot analyses. Anti-BosR rat polyclonal antibody α-BosR, anti-RpoS monoclonal antibody 6A7-101, anti-FlaB monoclonal antibody 8H3-33, or anti-OspC monoclonal antibody 1B2-105A were used, respectively, for detecting BosR, RpoS, FlaB, or OspC (Ouyang et al., 2009b, 2011). Immunoblots were developed colorimetrically using 4-chloro-1-napthol (Sigma) as the substrate or by chemiluminescence using ECL Plus Western Blotting Detection system (Thermo Fisher Scientific).

### Electrophoretic Mobility Shift Assay (EMSA)

A digoxigenin-labeled probe representing the BS2 site in the *rpoS* promoter of *B. burgdorferi* was prepared as previously described (Ouyang et al., 2011, 2014a). Binding reaction was carried out in a buffer containing 20 mM Hepes (pH 7.5), 50 μg/ml poly[d(A-T)], 5% (w/v) glycerol, 1 mM DTT, 100 μg/ml BSA, 1 mM MgCl_2_, and 50 mM KCl. For binding assays, various amounts of purified rBosR were incubated with 30 fmol of a digoxigenin-labeled probe at 37°C for 30 min. The reaction mixtures were then loaded on a 5% native polyacrylamide gel and resolved at 90 V, 4°C for 90 min in Tris borate-EDTA buffer (45 mM Tris, 45 mM boric acid, 1 mM EDTA, pH 7.5). After electrophoresis, DNA was transferred onto a positively-charged Nylon membrane (Roche Applied Science, Penzberg, Germany) by electroblotting, and fixed by UV cross linking. The digoxigenin-labeled probes were subsequently detected by enzyme immunoassays using an antibody (anti-digoxigenin-AP, Fab fragments) and the chemiluminescent substrate disodium 3-(4-methoxyspiro {l,2-dioxetane-3,2′-(5′-chloro)tricyclo[3.3.1.1^3, 7^]decan}-4-yl) phenyl phosphate (CSPD) (Roche Applied Science). The membrane was exposed to the Fujifilm LAS-3000 Imager (Fujifilm), and images were analyzed by using the MultiGauge V3.0 software (Fujifilm).

### Statistical Analysis

Data of metal content analyses were expressed as the mean ± standard deviation (SD), and analyzed by using an unpaired Student's *t* test, in which statistical significance was determined when *P* < 0.05.

## Results

### BosR Contains Two CXXC Motifs in its C-terminal Dimerization Domain

**Figure 1:**
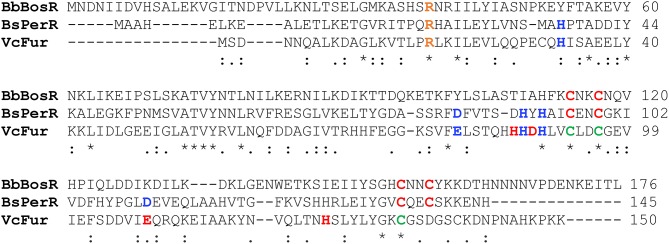
Alignment of BosR amino acid sequences with *V. cholera* Fur (VcFur) and *B. subtilis* PerR (BsPerR) by using Clustal Omega. Asterisks, periods, and colons represent completely conserved residues, conserved substitutions, and semi-conserved substitutions, respectively. Residues consisting the Zn-binding structural site (S1) are indicated in red, while residues comprising the metal-sensing regulatory site (S2) are indicated in blue. The conserved cysteine residues in VcFur are shown in green. R39, indicated in orange, has been shown to be important for BosR function.

BosR has homology to the *Vibrio cholerae* Fur protein (VcFur) and the *Bacillus subtilis* PerR protein (BsPerR) (Fraser et al., 1997; Boylan et al., 2003; Katona et al., 2004; Ouyang et al., 2011). To decipher potential structural motifs and/or amino acid residues strategic for metal binding and the function of BosR, we performed an amino acid sequence alignment of BosR and its homologs. Compared with VcFur and BsPerR, BosR shares 22.5 and 21.8% identity, and 43 and 49% similarity, respectively. Based on the alignment, there are seventeen residues that are completely conserved among three proteins. Among these completely conserved residues, two cysteines (C114, C117) in the putative dimerization domain of BosR constitute a potential CXXC motif (Figure 1). In addition, BosR contains another CXXC motif in its dimerization domain, consisting of the completely conserved cysteine (C153) and another cysteine (C156) (Figure 1). The dimerization domain of BsPerR also contains two CXXC motifs including a completely conserved C96XXC99 motif and a partially conserved C136XXC139 motif, whereas VcFur contains only three conserved cysteine residues (i.e., C93, C96, C133) among which C93 and C96 constitute a CXXC motif (Figure 1). Studies have shown that the two CXXC motifs of BsPerR constitute a tetrahedral ZnS_4_ S1 site critical for Zn coordination and protein function (Lee and Helmann, 2006; Traoré et al., 2006, 2009; Jacquamet et al., 2009). In contrast, the CXXC motif of VcFur is not involved in metal binding and gene regulation (Sheikh and Taylor, 2009). Heretofore, the functions of the putative CXXC motifs in BosR remain unaddressed. To study the potential roles of these two structural motifs, we introduced point mutations into four cysteines.

### Purification of Recombinant BosR With a StrepII-tag

**Figure 2:**
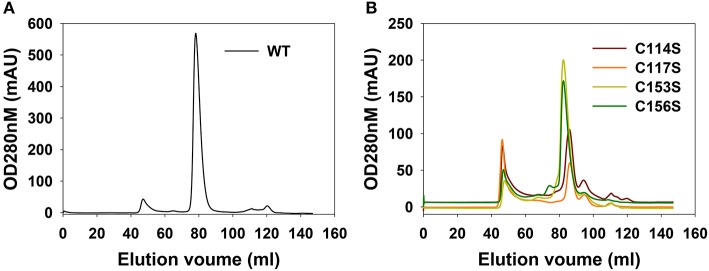
FPLC purification of recombinant BosR **(A)** and mutant proteins **(B)**. A representative chromatogram on a Superdex 200 column from three biological replicates is shown.

Previously, we created a BosR overexpression construct pOY73 in which BosR was fused with a N-terminal His6–SUMO tag (Ouyang et al., 2011). We herein generated another BosR overexpression construct pOY436, in which a StrepII-tag was added to the C-terminal of BosR. Compared with the His6 tag, the StrepII-tag does not react with heavy metal ions such as Cu, Ni, Zn and Co (Schmidt and Skerra, 2007), which excludes potential metal depletion of rBosR during protein purification. As shown in Figure 2A, after Strep-Tactin affinity purification and FPLC, pure rBosR was obtained. Specifically, in the Superdex 200 FPLC purification of rBosR, a sharp peak was observed with a peak maximum (the elution volume) of 78.79 ml. When the molecular weight (MW) of rBosR was calculated based on the gel filtration chromatogram, rBosR was estimated to have a MW of ~44.17 kDa. Given that the theoretical MW of rBosR-StrepII is 21.25 kDa, our data support that rBosR-StrepII eluted predominantly as a dimer.

### Critical Roles of the CXXC Motifs in BosR Dimerization

To determine the roles of CXXC motifs in the dimerization of BosR, we introduced point mutations into the BosR overexpression construct pOY436. Specifically, four BosR point mutants including C114S, C117S, C153S, or C156S were created through site-directed mutagenesis. rBosR variants were also overexpressed and purified via affinity purification and FPLC. As shown in Figure 2B, when the first C114XXC117 motif of BosR was mutated, C114S or C117S rBosR showed a peak maximum of ~86.23 or ~86.29 ml, respectively, in FPLC. When MW of these proteins were calculated, C114S or C117S rBosR had a MW of ~26.26 or ~25.72 kDa, respectively, suggesting these two proteins eluted predominantly as monomer. When the second C153XXC156 motif of BosR was mutated, C153S or C156S rBosR showed a peak maximum of ~82.68 or ~82.47 ml, respectively, in FPLC (Figure 2B). Based on the FPLC, C153S or C156S rBosR had a MW of ~35.82 or ~35.99 kDa. These data indicate that mutation of the CXXC motifs of BosR significantly affected the ability of BosR to form dimers.

### The CXXC Motifs Are Involved in the Binding of Zn

**Figure 3:**
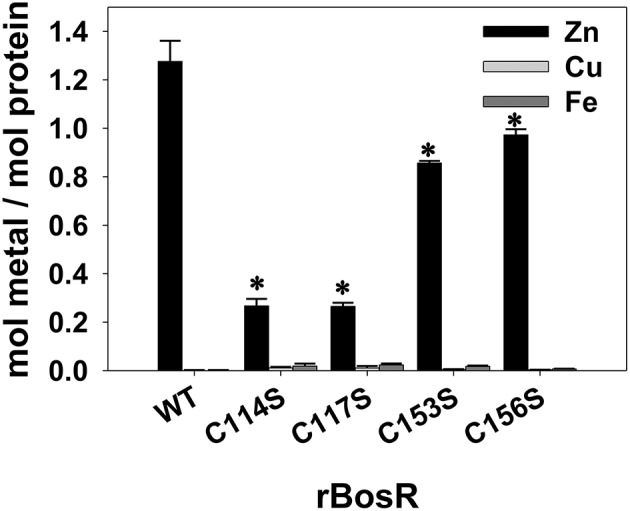
Metal content analysis of the recombinant BosR and variants. Proteins from at least three independent FPLC purifications were used for analyses. Protein concentration was determined by using the BCA protein assay. Metal content was determined by ICP-AES as detailed in Materials and Methods. Data are presented as the mean values ± standard deviations. The asterisk denotes statistical significance using Student's *t* test (*P* <0.05) when compared with WT rBosR. The graph shows only the values of Zn, Cu, and Fe, and the concentrations of Mn, Cr, Cd, Ni, Mg, and Pb are below the detection limit.

To determine the metal ions bound by rBosR-StrepII, metal content was analyzed by using inductively coupled plasma atomic emission spectrometry (ICP-AES). Our data showed that WT rBosR and rBosR variants (i.e., C114S, C117S, C153S, C156S) did not contain detectable levels of Mn (<0.001 ppm), Cr (<0.001 ppm), Cd (<0.001 ppm), Ni (<0.002 ppm), Mg (<0.003 ppm), or Pb (<0.003 ppm). Moreover, 0.001 ppm Fe or Cu (i.e., ~0.002 mol Fe or Cu per mol of protein) was detected in WT rBosR-StrepII (Figure 3); the limit of detection for these two ions in current experiment is 0.001 ppm, suggesting that Fe and Cu were almost undetectable in rBosR.

We also measured the level of Zn in rBosR-StrepII. As shown in Figure 3, WT rBosR-StrepII contained ~1.3 mol of Zn per mol of protein (Figure 3). Our data also showed that mutation of the CXXC motifs of BosR significantly impaired the ability of BosR to bind Zn. As shown in Figure 3, C114S or C117S rBosR contained ~0.27 mol of Zn per mol of protein, whereas ~0.86 or ~0.97 mol of Zn per mol of protein was detected in C153S or C156S rBosR, respectively (Figure 3). These data strongly suggest that the CXXC motifs, particularly the first C114XXC117 motif, are involved in the binding of Zn.

### The CXXC Motifs Are Essential for BosR Function *in vivo*

**Figure 4:**
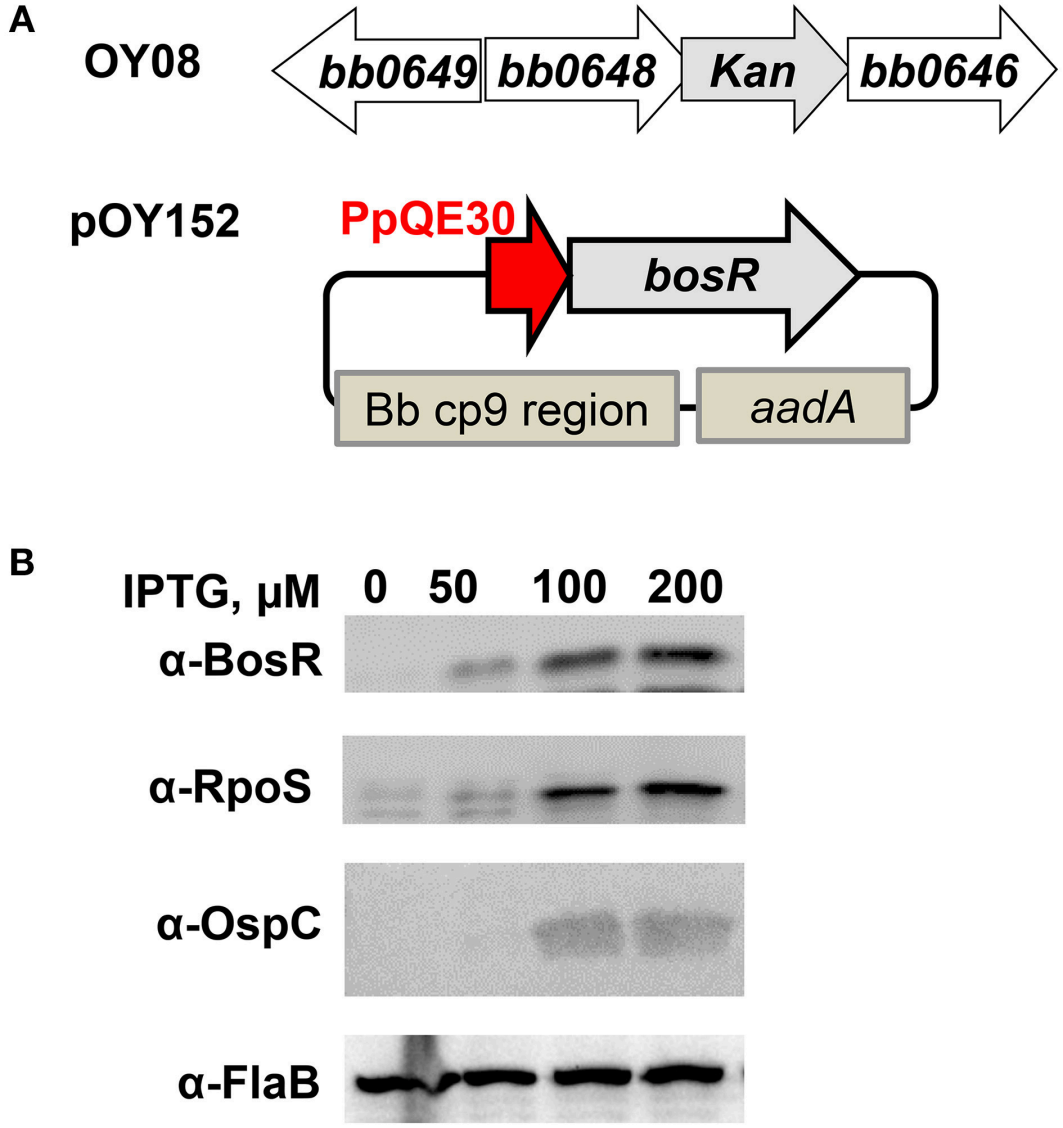
*Trans*-complementation of the *bosR* mutant OY08. To complement *bosR* mutation in *trans*-, a shuttle vector pOY152 in which *bosR* expression was directly controlled by the IPTG-inducible promoter PpQE30 was created. Then pOY152 was transformed into the *bosR* mutant OY08, resulting in strain OY110. **(A)** Schematic representations of the *bosR* mutant and the shuttle vector pOY152. In the mutant OY08, the *bosR* (*bb0647*) ORF was replaced with a kanamycin resistance cassette (kan). The shuttle vector pOY152 contains three essential elements including PpQE30-*bosR*, Bb cp9 region, and PflgB-*aadA*. Bb cp9 region represents the three open reading frames of *B. burgdorferi* cp9 plasmid that ensure autonomous replication of the shuttle vector in *B. burgdorferi*. *aadA* encodes streptomycin/spectinomycin adenylyltransferase and confers streptomycin resistance in *B. burgdorferi*. **(B)** Gene expression in *B. burgdorferi* was assessed by immunoblot. Strain OY110 was grown in BSK-II medium containing varying concentrations of IPTG and cells were harvested at late log-phase (~10^8^ cells ml^−1^). Gene expression was assessed by immunoblot. Approximately 4 × 10^7^ spirochetes were loaded onto each lane. Concentrations of IPTG are indicated above the image. Specific antibodies, denoted as α-, are indicated on the left. FlaB was used as a normalization control for equivalent loading. For detection of FlaB, RpoS, and OspC, cell lysate from WT stain 297 was included as a control. Immunoblots for BosR, RpoS, and FlaB detection were developed by chemiluminescence, whereas immunoblots for OspC detection were developed colorimetrically. Shown are representative images from 2 to 4 independent experiments with at least two biological replicates.

To investigate the influence of the CXXC motifs on the regulatory function of BosR, a genetic *trans*-complementation approach was employed in this study. To this end, an IPTG-inducible *bosR* expression construct (pOY152) was created, in which *bosR* transcription was directly controlled by the IPTG-inducible PpQE30 promoter (Figure 4A). Plasmid pOY152 was then introduced into our *bosR* mutant OY08 through electroporation, generating OY110. Strain OY110 was cultivated in BSK-II medium containing various amounts of IPTG (i.e., 0-, 50-, 100-, or 200-μM). When bacterial growth reached late-log phase, spirochetes were harvested, and protein production was analyzed through immunoblot. As shown in Figure 4B, BosR synthesis was induced by IPTG in a dose-dependent manner. As BosR activates the expression of *rpoS* in *B. burgdorferi*, we also examined the levels of RpoS and RpoS-dependent OspC in OY110. As shown in Figure 4B, consistent with the production of BosR, syntheses of RpoS and OspC were also induced by IPTG in a dose-dependent manner. These data not only confirmed our previous observation that BosR activates *rpoS* expression, but also set the stage for further strategic use of this *trans*-complementation approach in investigating the contributions of BosR's potential structural motifs and key residues.

**Figure 5:**
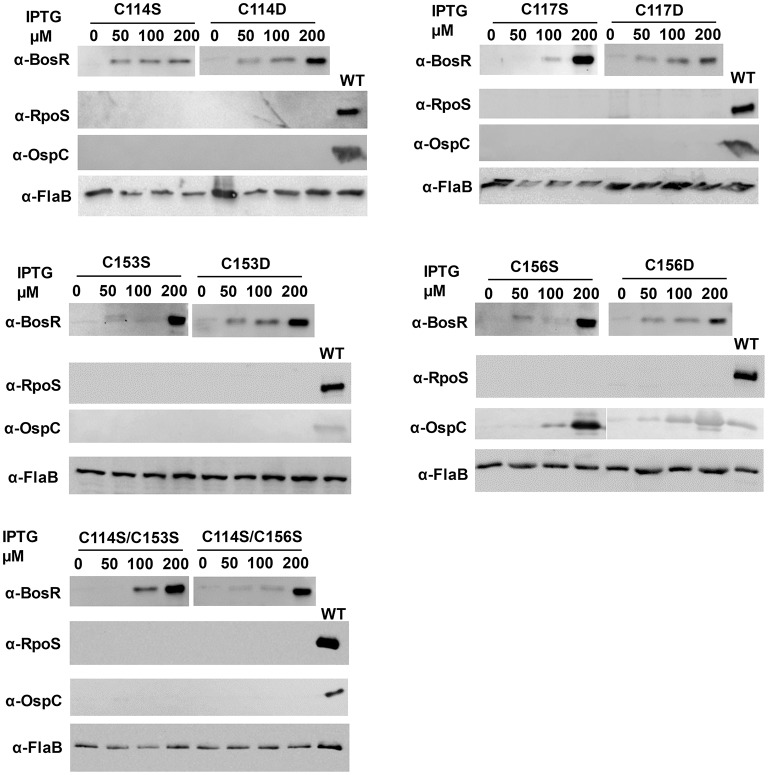
The CXXC motifs are essential for BosR to activate *rpoS* expression. To mutate the CXXC motifs, specific point mutation was introduced into *bosR* in pOY152. The resultant shuttle vectors were introduced into the *bosR* mutant OY08. Spirochetes grown in BSK-II medium containing varying concentrations of IPTG were harvested at late log-phase (~10^8^ cells ml^−1^). *B. burgdorferi* whole cell lysates were analyzed by immunoblot. Approximately 4 × 10^7^ spirochetes were loaded onto each lane. Concentrations of IPTG and BosR mutant names (e.g., C114S, etc.) are indicated above the image. Specific antibodies, denoted as α-, are indicated on the left. FlaB was used as a normalization control for equivalent loading. For detection of FlaB, RpoS, and OspC, cell lysate from WT stain 297 was included as a control. Immunoblots for BosR, RpoS, and FlaB detection were developed by chemiluminescence, whereas immunoblots for OspC detection were developed colorimetrically. Shown are representative images from 2 to 4 independent experiments with at least two biological replicates.

Next, residues C114, C117, C153, and C156 were individually replaced by serine or aspartic acid. Specifically, point mutations of BosR including C114S, C114D, C117S, C117D, C153S, C153D, C156S, and C156D were introduced into the construct pOY152 via site-directed mutagenesis. These mutated constructs were then transformed into the *bosR* mutant OY08, in order to assess whether mutated BosR variants are capable of complementing *bosR* mutation and activating expression of *rpoS* and *ospC*. *B. burgdorferi* strains were also cultivated in the presence of various amounts of IPTG (i.e., 0-, 50-, 100-, or 200-μM). Late log-phase cells were harvested and analyzed by immunoblot. As shown in Figure 5, syntheses of BosR variants were induced by IPTG in all these strains. However, in cells producing BosR variants, RpoS was not detected, suggesting that these BosR mutants were incapable of complementing *bosR* mutation and activating *rpoS* expression. In cells producing C114S, C114D, C117S, C117D, C153S, or C153D BosR variants, OspC was undetected. However, IPTG-dose dependent induction of OspC was observed in cells producing C156S or C156D BosR variants (Figure 5).

We also mutated both CXXC motifs in BosR and analyzed its impact on gene expression. Specifically, double point mutations (i.e., C114S/C153S and C114S/C156S) were created in pOY152. Mutated constructs were also introduced into the *bosR* mutant. As shown in Figure 5, increasing levels of BosR variants were induced by IPTG in strains harboring these two double mutations. However, production of RpoS or OspC was not detected in these strains.

### Mutation of the CXXC Motifs Abolishes Binding of BosR to the *rpoS* Promoter

**Figure 6:**
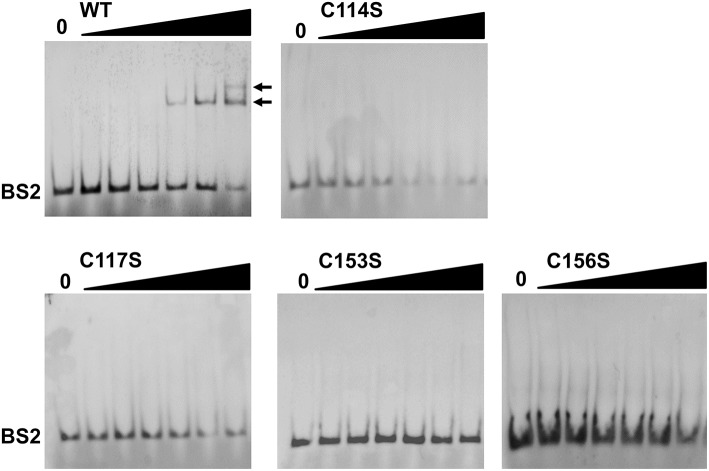
BosR, but not CXXC mutant proteins, binds to the *rpoS* promoter. EMSA was conducted as described in Materials and Methods. Nucleotides representing the BS2 site of the *rpoS* promoter was synthesized and labeled with digoxigenin. In a typical EMSA, 30 fmol labeled BS2 probe was incubated with varying amounts of BosR at 37°C for 30 min. Increasing concentrations of protein (10-, 20-, 50-, 100-, 200-, and 500-nM) are designated by black triangles, while the reactions contain no protein are indicated by “0.” Protein name is indicated above the image. Bound DNA is denoted by arrows. Shown are representative images from at least two independent experiments.

Previous studies (Ouyang et al., 2011, 2014a; Katona, 2015) have established that BosR directly activates *rpoS* expression by binding to the *rpoS* promoter region. More specifically, BosR binds with high affinity to a site called BS2 overlapping with the −24/−12 *rpoS* promoter. To understand why BosR CXXC mutants failed to activate *rpoS* expression, EMSAs were also employed in this study to assess the binding of BosR variants to the BS2 site. As shown in Figure 6, when 100-, 200-, or 500-nM WT rBosR-StrepII was incubated with the probe representing BS2, DNA shift was observed, suggesting that WT rBosR-StrepII binds to the BS2 site. However, when rBosR (C114S, C117S, C153S, or C156S) variants were incubated with BS2, DNA shift was not observed, demonstrating that these BosR mutants were incapable of binding to the *rpoS* promoter.

## Discussion

Structure-function analyses of numerous Fur proteins have revealed that Fur binds its target DNA as a dimer. Protein dimerization requires Zn, which is coordinated via the structural S1 site in the dimerization domain of Fur proteins. To date, it has been clearly demonstrated that different geometry and amino acid residues are used by Fur protein for coordinating Zn in the S1 site (Pohl et al., 2003; Lee and Helmann, 2006; Pecqueur et al., 2006; Traoré et al., 2006, 2009; Lucarelli et al., 2007; Ahmad et al., 2009; An et al., 2009; Carpenter et al., 2009; Jacquamet et al., 2009; Sheikh and Taylor, 2009; Davies et al., 2011; Dian et al., 2011; Shin et al., 2011; Butcher et al., 2012; Makthal et al., 2013; Troxell and Hassan, 2013; Fillat, 2014; Gilston et al., 2014; Lin et al., 2014; Deng et al., 2015). For instance, for *E. coli* Fur protein, the first member of the Fur family characterized, its S1 site consists of two cysteines (i.e., C92, C95) in the dimerization domain (Fillat, 2014; Seo et al., 2014). Moreover, in BsPerR, four cysteines (i.e., C96, C99, C136, and C139) in the dimerization domain form two CXXC motifs and constitute a tetrahedral Zn(Cys)_4_ structural site (Lee and Helmann, 2006; Traoré et al., 2006, 2009; Jacquamet et al., 2009). Previous analyses have indicated that BosR binds Zn and protein binds its DNA targets as a dimer (Boylan et al., 2003; Katona et al., 2004; Seshu et al., 2004; Samuels and Radolf, 2009; Ouyang et al., 2011, 2014a, 2016; Wang et al., 2013, 2017; Shi et al., 2014; Katona, 2015). However, it remained unknown what residues or structural motifs of BosR coordinate Zn and contribute to protein dimerization. When BosR was aligned with its homolog BsPerR, we found that BosR also contained four cysteines forming two putative CXXC motifs in its dimerization domain. However, the presence of the CXXC motifs does not necessarily mean they are involved in Zn binding. Indeed, in VcFur, another homolog of BosR, the C92XXC95 motif is irrelevant to the Zn-coordinating structural S1 site and protein regulatory function (Sheikh and Taylor, 2009). Rather, the S1 site of VcFur consists of four residues (i.e., H87, D89, E108, and H125) located in both DNA-binding domain and dimerization domain. To gain insight into whether the CXXC motifs impact metal binding as well as multiple aspects of BosR function, we introduced mutations into these two motifs. Molecular analyses and phenotypic characterization of the mutants clearly demonstrate that these two motifs of BosR constitute the potential structural S1 site for Zn coordination. This conclusion was drawn based on several lines of evidence. First, our data demonstrate that both CXXC motifs of BosR are involved in the binding of Zn. When the C114XXC117 motif was mutated, binding of Zn was significantly diminished (~80%). When the second C153XXC156 motif was mutated, the capacity of BosR to bind Zn was also impaired (~34%). Our analyses also revealed that BosR does not bind Mn, Mg, Cr, Cd, Ni, or Pb. Regarding the binding of Fe and Cu, only ~0.002 mol Fe or Cu was detected in one mol of WT BosR, implying that BosR likely does not bind these two metal ions. These results are different from a previous study reporting that BosR is capable of binding Fe and Cu (Wang et al., 2017). This discrepancy may relate to the strategically different experimental approaches used in these two studies. In the present work, purified rBosR (functional in binding to the *rpoS* promoter) was directly used for metal analyses, whereas the other study (Wang et al., 2017) first incubated rBosR with unusually excess amount of Fe or Cu (at a molar ratio of eight metal ions per BosR dimer) before protein metal analyses. Moreover, our data support that both CXXC motifs of BosR contribute to protein dimerization. In FPLC purification, WT rBosR was predominantly eluted as a dimer. However, when C114 or C117 was mutated, rBosR mutant was purified predominantly as a monomer, suggesting that the C114XXC117 motif is essential for the dimerization of BosR. When analyzing the FPLC chromatogram and molecule weight of C153S rBosR and C156S rBosR, our data suggest that the C153XXC156 motif also contribute to the dimerization of BosR. Of note, based on FPLC chromatogram, C153S or C156S rBosR was estimated to have a MW of ~35.82 or ~35.99 kDa, respectively. The reason for this observation remains unknown. One possibility is that these rBosR exist in solution in a monomer-dimer equilibrium. As indicated in Figure 2, an obvious peak representing protein aggregates was observed in the FPLC chromatogram of mutant rBosR, suggesting that the solubility of these mutated proteins was decreased. Therefore, mutation of C153 or C156 may cause unknown protein conformational changes which in turn, changes the migration of BosR in FPLC. Future structure analyses of rBosR and its mutants via circular dichroism spectroscopy and/or X-ray crystallization may provide an answer to this question.

It is worth noting that the two CXXC motifs of BosR do not have equal contributions to protein properties and function. Relative to the C153XXC156 motif, the C114XXC117 motif appears to have greater impact on protein dimerization. Likewise, mutation of the C153XXC156 motif partially (~34%) affected Zn binding, while mutation of the C114XXC117 motif resulted in pronounced (~80%) decrease of Zn binding. Despite their different impacts on protein dimerization and Zn binding, both CXXC motifs are required for the *in vivo* regulatory function of BosR. This assertion is strongly supported by our data presented in the current study. First, all mutated proteins do not bind to the *rpoS* promoter in EMSAs. More importantly, in a functional *trans*-complementation study, BosR with either CXXC motif mutated failed to complement a *B. burgdorferi bosR* mutant and restore *rpoS* expression. When WT BosR was produced in a *bosR* mutant, synthesis of RpoS was detected. In contrast, when BosR with mutation in any of the four cysteine residues was produced in the *bosR* mutant, RpoS synthesis was not detected. In *B. burgdorferi*, expression of *ospC* is dependent on RpoS (Hübner et al., 2001; Alverson et al., 2003; Caimano et al., 2004, 2005, 2007; Eggers et al., 2004; Yang et al., 2005; Gilbert et al., 2007; Ouyang et al., 2008, 2010; Drecktrah et al., 2013). Accordingly, induction of OspC synthesis was not detected in *B. burgdorferi* producing C114S, C114D, C117S, C117D, C153S, or C153D mutated BosR. Interestingly, induction of OspC production was observed in strains producing C156S or C156D BosR, whereas OspC was undetected in strains producing C114S/C156S double mutant BosR. The mechanism(s) for this aberrant *ospC* expression in strains producing C156S or C156D BosR hitherto remains unknown.

Our data presented in this study strongly support that the two CXXC motifs in the dimerization domain of BosR constitute the Zn-coordination structural S1 site. Both CXXC motifs of BosR and the four cysteine residues contained in the motifs are important for protein dimerization, Zn binding, and gene regulation. Similar observations have been made on the CXXC motifs of BsPerR, in which both C96XXC99 and C136XXC139 motifs are essential for Zn binding and gene regulation (Lee and Helmann, 2006; Traoré et al., 2006, 2009; Jacquamet et al., 2009). When C96S, C99S, C136S, or C139S mutation was introduced into BsPerR, the repression on *mgrA* expression by BsPerR was abolished (Lee and Helmann, 2006). Our findings thus support that the potential structural S1 site of BosR is more comparable to the one in BsPerR than the one in VcFur. Our study also prompts a number of new and important questions on the unknown structure-function relationship of BosR. For instance, how does the CXXC motifs in BosR coordinate Zn? How does the mutation of each cysteine residue influence the conformation and function of BosR? Aberrant σ^70^-dependent *ospC* expression was observed only in strains producing C156S or C156D BosR mutant, but not in strains producing C114S, C114D, C117S, C117D, C153S, or C153D BosR, suggesting clear different impacts of C156 on BosR structure/function than other cysteine residues. It also remains unknown how much Zn is required for the function of BosR. Based on the data presented in this current study, probably a fully Zn-loaded BosR active dimer is prerequisite for binding target DNA and gene regulation.

In the current study, we determined the contributions of two CXXC motifs of BosR to protein function via a genetic approach. This approach is applicable for future studies directed at understanding the protein structure-function relationship of BosR in the Lyme disease pathogen. BosR appears not to harbor a metal-sensing regulatory S2 site found in VcFur (consisting of four residues including H33, E81, H88, H90) (Sheikh and Taylor, 2009) or BsPerR (consisting of five residues including H37, D85, H91, H93, D104) (Traoré et al., 2006; Jacquamet et al., 2009) (Figure 1). Moreover, BosR lacks residues constituting the S3 site of *H. pylori* and *C. jejuni* Fur (Dian et al., 2011; Butcher et al., 2012). In fact, BosR contains only one residue (i.e., H111) that may be involved in metal coordination (other than the CXXC motifs) (Figure 1). This information suggests that BosR may lack a classical metal-responsive S2 regulatory site. More specifically, BosR may employ a unique mechanism(s) rather than metal sensing for the activation of its DNA binding activity. Genetic studies on the residues unique for BosR will be essential for gaining insight into the mechanistic details behind the function of BosR, particularly given the lack of a three-dimensional structure of BosR.

## Author Contributions

CM, XL, and SP performed the experiments. CM, XL, and ZO analyzed results. ZO designed the study and wrote the manuscript. All authors read and approved the manuscript.

### Conflict of Interest Statement

The authors declare that the research was conducted in the absence of any commercial or financial relationships that could be construed as a potential conflict of interest.
